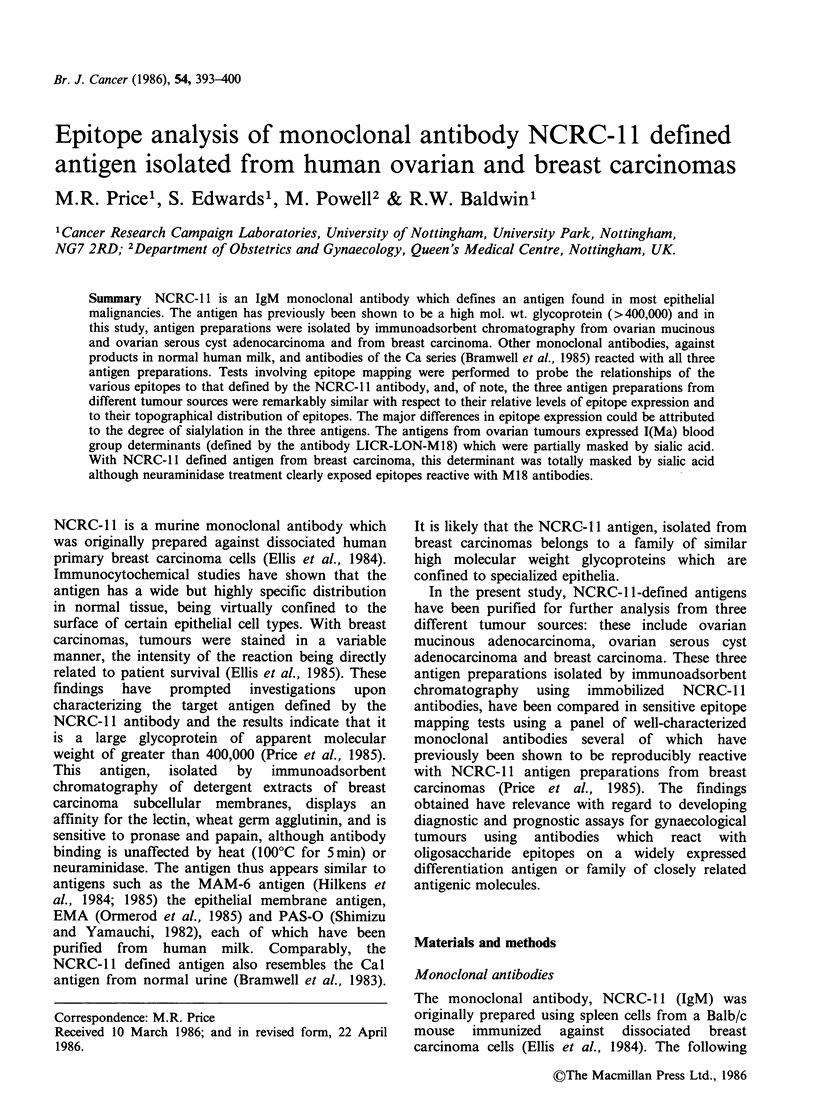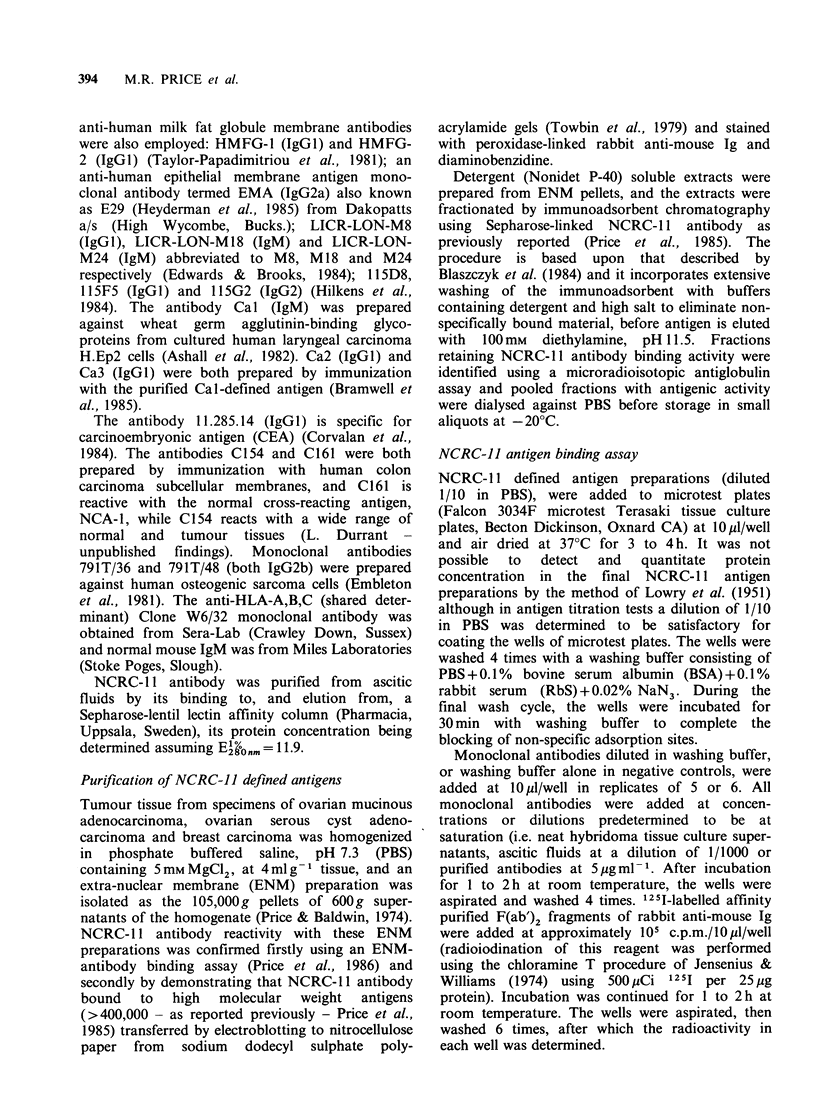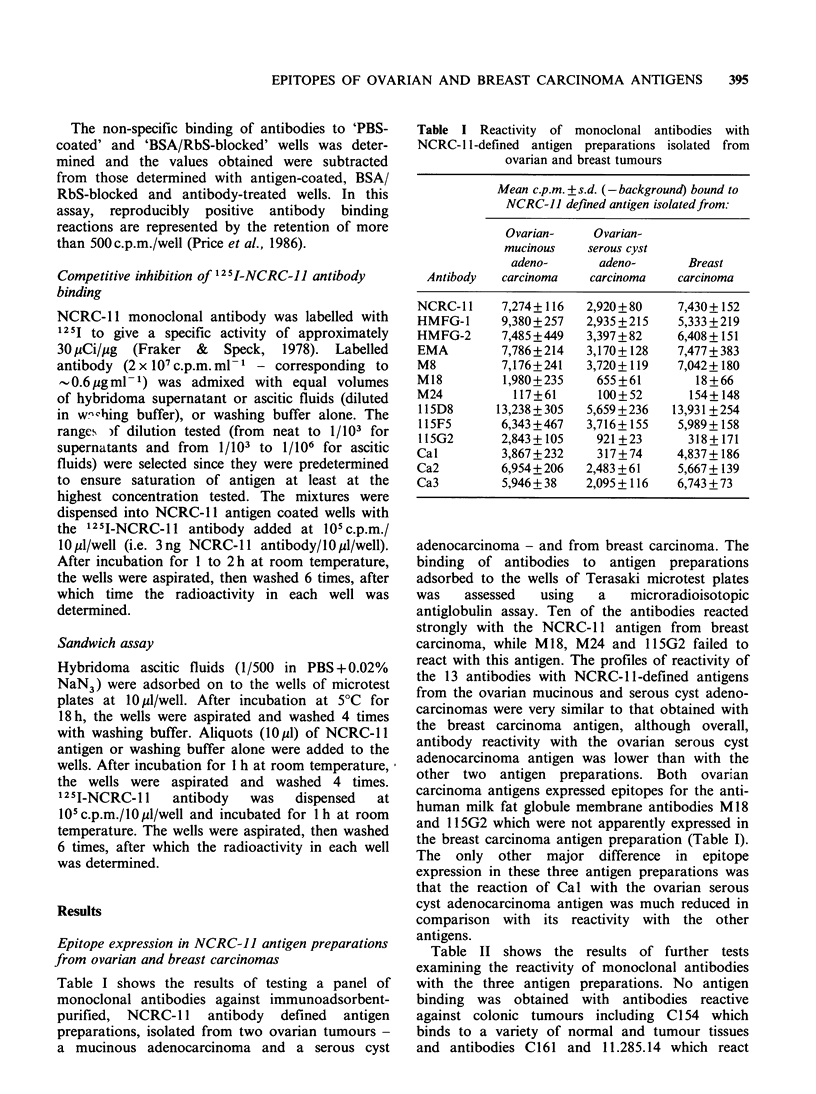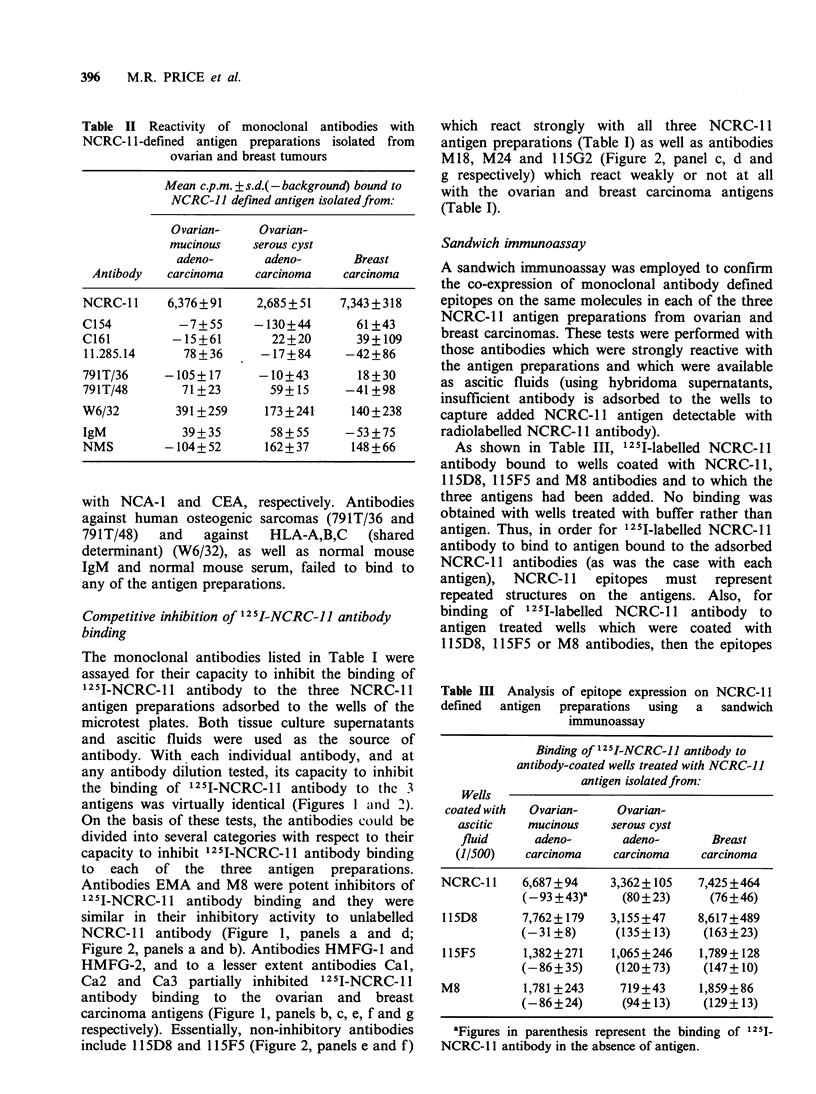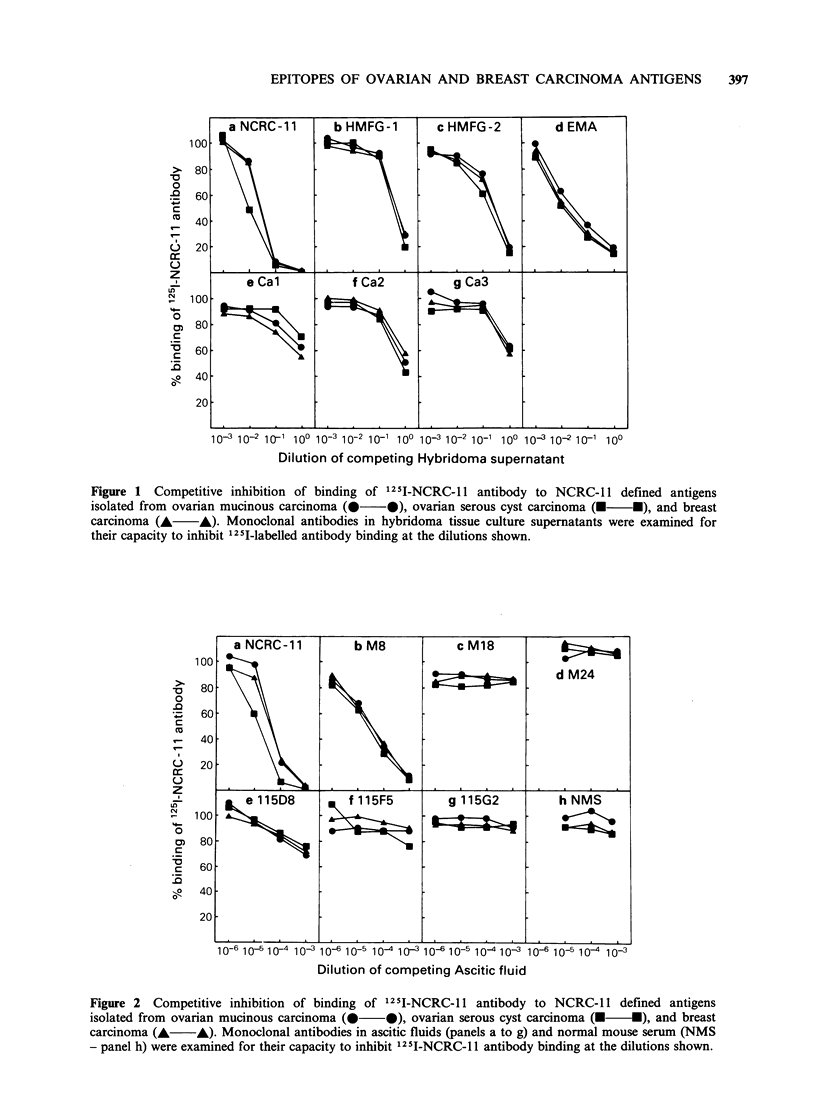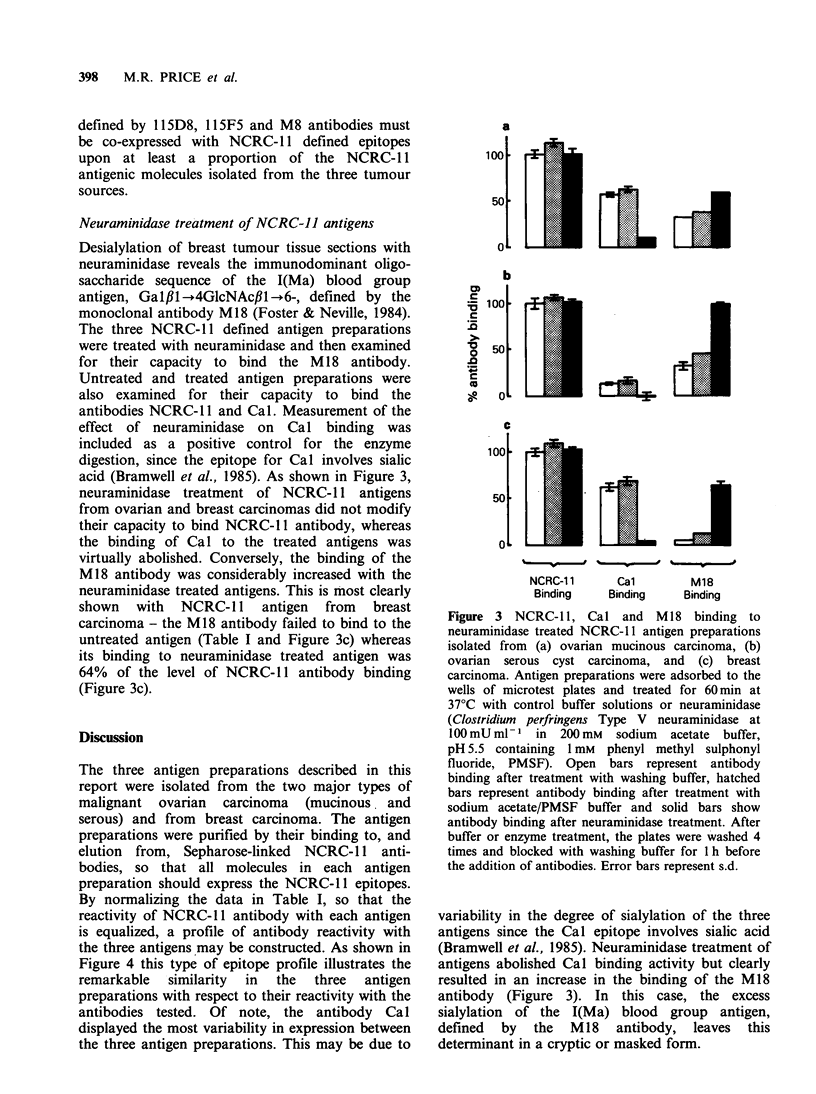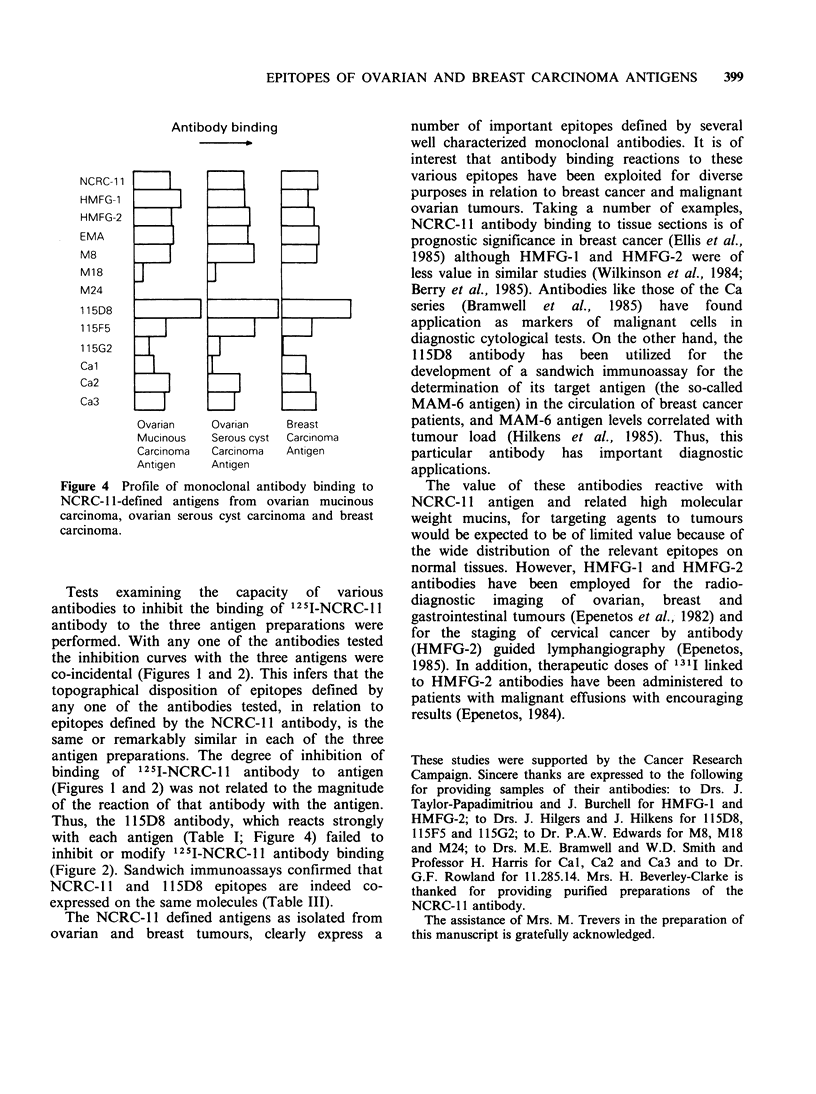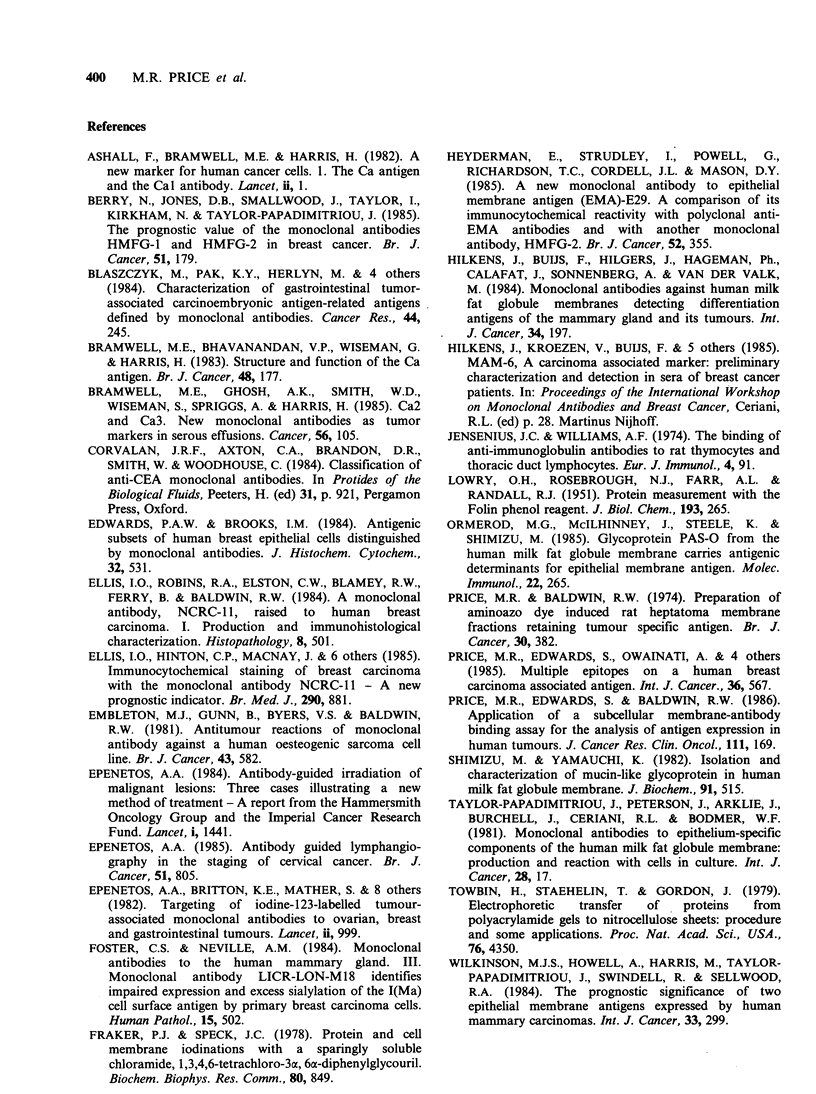# Epitope analysis of monoclonal antibody NCRC-11 defined antigen isolated from human ovarian and breast carcinomas.

**DOI:** 10.1038/bjc.1986.189

**Published:** 1986-09

**Authors:** M. R. Price, S. Edwards, M. Powell, R. W. Baldwin

## Abstract

NCRC-11 is an IgM monoclonal antibody which defines an antigen found in most epithelial malignancies. The antigen has previously been shown to be a high mol. wt. glycoprotein (greater than 400,000) and in this study, antigen preparations were isolated by immunoadsorbent chromatography from ovarian mucinous and ovarian serous cyst adenocarcinoma and from breast carcinoma. Other monoclonal antibodies, against products in normal human milk, and antibodies of the Ca series (Bramwell et al., 1985) reacted with all three antigen preparations. Tests involving epitope mapping were performed to probe the relationships of the various epitopes to that defined by the NCRC-11 antibody, and, of note, the three antigen preparations from different tumour sources were remarkably similar with respect to their relative levels of epitope expression and to their topographical distribution of epitopes. The major differences in epitope expression could be attributed to the degree of sialylation in the three antigens. The antigens from ovarian tumours expressed I(Ma) blood group determinants (defined by the antibody LICR-LON-M18) which were partially masked by sialic acid. With NCRC-11 defined antigen from breast carcinoma, this determinant was totally masked by sialic acid although neuraminidase treatment clearly exposed epitopes reactive with M18 antibodies.


*Br. J. Cancer* (1986), **54**, 393–400

### Epitope analysis of monoclonal antibody NCRC-11 defined antigen isolated from human ovarian and breast carcinomas

M.R. Price[1], S. Edwards[1], M. Powell[2] & R.W. Baldwin[1]

[1]*Cancer Research Campaign Laboratories, University of Nottingham, University Park, Nottingham, NG7 2RD;* [2]*Department of Obstetrics and Gynaecology, Queen's Medical Centre, Nottingham, UK.*

**Summary** NCRC-11 is an IgM monoclonal antibody which defines an antigen found in most epithelial malignancies. The antigen has previously been shown to be a high mol. wt. glycoprotein (>400,000) and in this study, antigen preparations were isolated by immunoadsorbent chromatography from ovarian mucinous and ovarian serous cyst adenocarcinoma and from breast carcinoma. Other monoclonal antibodies, against products in normal human milk, and antibodies of the Ca series (Bramwell *et al.*, 1985) reacted with all three antigen preparations. Tests involving epitope mapping were performed to probe the relationships of the various epitopes to that defined by the NCRC-11 antibody, and, of note, the three antigen preparations from different tumour sources were remarkably similar with respect to their relative levels of epitope expression and to their topographical distribution of epitopes. The major differences in epitope expression could be attributed to the degree of sialylation in the three antigens. The antigens from ovarian tumours expressed I(Ma) blood group determinants (defined by the antibody LICR-LON-M18) which were partially masked by sialic acid. With NCRC-11 defined antigen from breast carcinoma, this determinant was totally masked by sialic acid although neuraminidase treatment clearly exposed epitopes reactive with M18 antibodies.

NCRC-11 is a murine monoclonal antibody which was originally prepared against dissociated human primary breast carcinoma cells (Ellis *et al.*, 1984). Immunocytochemical studies have shown that the antigen has a wide but highly specific distribution in normal tissue, being virtually confined to the surface of certain epithelial cell types. With breast carcinomas, tumours were stained in a variable manner, the intensity of the reaction being directly related to patient survival (Ellis *et al.*, 1985). These findings have prompted investigations upon characterizing the target antigen defined by the NCRC-11 antibody and the results indicate that it is a large glycoprotein of apparent molecular weight of greater than 400,000 (Price *et al.*, 1985). This antigen, isolated by immunoadsorbent chromatography of detergent extracts of breast carcinoma subcellular membranes, displays an affinity for the lectin, wheat germ agglutinin, and is sensitive to pronase and papain, although antibody binding is unaffected by heat (100°C for 5 min) or neuraminidase. The antigen thus appears similar to antigens such as the MAM-6 antigen (Hilkens *et al.*, 1984; 1985) the epithelial membrane antigen, EMA (Ormerod *et al.*, 1985) and PAS-O (Shimizu and Yamauchi, 1982), each of which have been purified from human milk. Comparably, the NCRC-11 defined antigen also resembles the Ca1 antigen from normal urine (Bramwell *et al.*, 1983). It is likely that the NCRC-11 antigen, isolated from breast carcinomas belongs to a family of similar high molecular weight glycoproteins which are confined to specialized epithelia.

In the present study, NCRC-11-defined antigens have been purified for further analysis from three different tumour sources: these include ovarian mucinous adenocarcinoma, ovarian serous cyst adenocarcinoma and breast carcinoma. These three antigen preparations isolated by immunoadsorbent chromatography using immobilized NCRC-11 antibodies, have been compared in sensitive epitope mapping tests using a panel of well-characterized monoclonal antibodies several of which have previously been shown to be reproducibly reactive with NCRC-11 antigen preparations from breast carcinomas (Price *et al.*, 1985). The findings obtained have relevance with regard to developing diagnostic and prognostic assays for gynaecological tumours using antibodies which react with oligosaccharide epitopes on a widely expressed differentiation antigen or family of closely related antigenic molecules.

### Materials and methods

### *Monoclonal antibodies*

The monoclonal antibody, NCRC-11 (IgM) was originally prepared using spleen cells from a Balb/c mouse immunized against dissociated breast carcinoma cells (Ellis *et al.*, 1984). The following

Correspondence: M.R. Price
Received 10 March 1986; and in revised form, 22 April 1986.

©The Macmillan Press Ltd., 1986

anti-human milk fat globule membrane antibodies were also employed: HMFG-1 (IgG1) and HMFG-2 (IgG1) (Taylor-Papadimitriou *et al.*, 1981); an anti-human epithelial membrane antigen monoclonal antibody termed EMA (IgG2a) also known as E29 (Heyderman *et al.*, 1985) from Dakopatts a/s (High Wycombe, Bucks.); LICR-LON-M8 (IgG1), LICR-LON-M18 (IgM) and LICR-LON-M24 (IgM) abbreviated to M8, M18 and M24 respectively (Edwards & Brooks, 1984); 115D8, 115F5 (IgG1) and 115G2 (IgG2) (Hilkens *et al.*, 1984). The antibody Ca1 (IgM) was prepared against wheat germ agglutinin-binding glycoproteins from cultured human laryngeal carcinoma H.Ep2 cells (Ashall *et al.*, 1982). Ca2 (IgG1) and Ca3 (IgG1) were both prepared by immunization with the purified Ca1-defined antigen (Bramwell *et al.*, 1985).

The antibody 11.285.14 (IgG1) is specific for carcinoembryonic antigen (CEA) (Corvalan *et al.*, 1984). The antibodies C154 and C161 were both prepared by immunization with human colon carcinoma subcellular membranes, and C161 is reactive with the normal cross-reacting antigen, NCA-1, while C154 reacts with a wide range of normal and tumour tissues (L. Durrant – unpublished findings). Monoclonal antibodies 791T/36 and 791T/48 (both IgG2b) were prepared against human osteogenic sarcoma cells (Embleton *et al.*, 1981). The anti-HLA-A,B,C (shared determinant) Clone W6/32 monoclonal antibody was obtained from Sera-Lab (Crawley Down, Sussex) and normal mouse IgM was from Miles Laboratories (Stoke Poges, Slough).

NCRC-11 antibody was purified from ascitic fluids by its binding to, and elution from, a Sepharose-lentil lectin affinity column (Pharmacia, Uppsala, Sweden), its protein concentration being determined assuming $E_{280\,nm}^{1\%} = 11.9$.

*Purification of NCRC-11 defined antigens*

Tumour tissue from specimens of ovarian mucinous adenocarcinoma, ovarian serous cyst adenocarcinoma and breast carcinoma was homogenized in phosphate buffered saline, pH 7.3 (PBS) containing 5 mM $MgCl_2$, at 4 ml $g^{-1}$ tissue, and an extra-nuclear membrane (ENM) preparation was isolated as the 105,000 *g* pellets of 600 *g* supernatants of the homogenate (Price & Baldwin, 1974). NCRC-11 antibody reactivity with these ENM preparations was confirmed firstly using an ENM-antibody binding assay (Price *et al.*, 1986) and secondly by demonstrating that NCRC-11 antibody bound to high molecular weight antigens (>400,000 – as reported previously – Price *et al.*, 1985) transferred by electroblotting to nitrocellulose paper from sodium dodecyl sulphate polyacrylamide gels (Towbin *et al.*, 1979) and stained with peroxidase-linked rabbit anti-mouse Ig and diaminobenzidine.

Detergent (Nonidet P-40) soluble extracts were prepared from ENM pellets, and the extracts were fractionated by immunoadsorbent chromatography using Sepharose-linked NCRC-11 antibody as previously reported (Price *et al.*, 1985). The procedure is based upon that described by Blaszczyk *et al.* (1984) and it incorporates extensive washing of the immunoadsorbent with buffers containing detergent and high salt to eliminate non-specifically bound material, before antigen is eluted with 100 mM diethylamine, pH 11.5. Fractions retaining NCRC-11 antibody binding activity were identified using a microradioisotopic antiglobulin assay and pooled fractions with antigenic activity were dialysed against PBS before storage in small aliquots at −20°C.

*NCRC-11 antigen binding assay*

NCRC-11 defined antigen preparations (diluted 1/10 in PBS), were added to microtest plates (Falcon 3034F microtest Terasaki tissue culture plates, Becton Dickinson, Oxnard CA) at 10 $\mu$l/well and air dried at 37°C for 3 to 4 h. It was not possible to detect and quantitate protein concentration in the final NCRC-11 antigen preparations by the method of Lowry *et al.* (1951) although in antigen titration tests a dilution of 1/10 in PBS was determined to be satisfactory for coating the wells of microtest plates. The wells were washed 4 times with a washing buffer consisting of PBS + 0.1% bovine serum albumin (BSA) + 0.1% rabbit serum (RbS) + 0.02% $NaN_3$. During the final wash cycle, the wells were incubated for 30 min with washing buffer to complete the blocking of non-specific adsorption sites.

Monoclonal antibodies diluted in washing buffer, or washing buffer alone in negative controls, were added at 10 $\mu$l/well in replicates of 5 or 6. All monoclonal antibodies were added at concentrations or dilutions predetermined to be at saturation (i.e. neat hybridoma tissue culture supernatants, ascitic fluids at a dilution of 1/1000 or purified antibodies at 5 $\mu g\,ml^{-1}$. After incubation for 1 to 2 h at room temperature, the wells were aspirated and washed 4 times. $^{125}$I-labelled affinity purified $F(ab')_2$ fragments of rabbit anti-mouse Ig were added at approximately $10^5$ c.p.m./10 $\mu$l/well (radioiodination of this reagent was performed using the chloramine T procedure of Jensenius & Williams (1974) using 500 $\mu$Ci $^{125}$I per 25 $\mu$g protein). Incubation was continued for 1 to 2 h at room temperature. The wells were aspirated, then washed 6 times, after which the radioactivity in each well was determined.

The non-specific binding of antibodies to 'PBS-coated' and 'BSA/RbS-blocked' wells was determined and the values obtained were subtracted from those determined with antigen-coated, BSA/RbS-blocked and antibody-treated wells. In this assay, reproducibly positive antibody binding reactions are represented by the retention of more than 500 c.p.m./well (Price *et al.*, 1986).

*Competitive inhibition of $^{125}$I-NCRC-11 antibody binding*

NCRC-11 monoclonal antibody was labelled with $^{125}$I to give a specific activity of approximately 30 $\mu$Ci/$\mu$g (Fraker & Speck, 1978). Labelled antibody ($2 \times 10^7$ c.p.m. ml$^{-1}$ – corresponding to $\sim$0.6 $\mu$g ml$^{-1}$) was admixed with equal volumes of hybridoma supernatant or ascitic fluids (diluted in washing buffer), or washing buffer alone. The ranges of dilution tested (from neat to 1/10$^3$ for supernatants and from 1/10$^3$ to 1/10$^6$ for ascitic fluids) were selected since they were predetermined to ensure saturation of antigen at least at the highest concentration tested. The mixtures were dispensed into NCRC-11 antigen coated wells with the $^{125}$I-NCRC-11 antibody added at 10$^5$ c.p.m./10 $\mu$l/well (i.e. 3 ng NCRC-11 antibody/10 $\mu$l/well). After incubation for 1 to 2 h at room temperature, the wells were aspirated, then washed 6 times, after which time the radioactivity in each well was determined.

*Sandwich assay*

Hybridoma ascitic fluids (1/500 in PBS+0.02% $NaN_3$) were adsorbed on to the wells of microtest plates at 10 $\mu$l/well. After incubation at 5°C for 18 h, the wells were aspirated and washed 4 times with washing buffer. Aliquots (10 $\mu$l) of NCRC-11 antigen or washing buffer alone were added to the wells. After incubation for 1 h at room temperature, the wells were aspirated and washed 4 times. $^{125}$I-NCRC-11 antibody was dispensed at 10$^5$ c.p.m./10 $\mu$l/well and incubated for 1 h at room temperature. The wells were aspirated, then washed 6 times, after which the radioactivity in each well was determined.

### Results

*Epitope expression in NCRC-11 antigen preparations from ovarian and breast carcinomas*

Table I shows the results of testing a panel of monoclonal antibodies against immunoadsorbent-purified, NCRC-11 antibody defined antigen preparations, isolated from two ovarian tumours – a mucinous adenocarcinoma and a serous cyst adenocarcinoma – and from breast carcinoma. The binding of antibodies to antigen preparations adsorbed to the wells of Terasaki microtest plates was assessed using a microradioisotopic antiglobulin assay. Ten of the antibodies reacted strongly with the NCRC-11 antigen from breast carcinoma, while M18, M24 and 115G2 failed to react with this antigen. The profiles of reactivity of the 13 antibodies with NCRC-11-defined antigens from the ovarian mucinous and serous cyst adenocarcinomas were very similar to that obtained with the breast carcinoma antigen, although overall, antibody reactivity with the ovarian serous cyst adenocarcinoma antigen was lower than with the other two antigen preparations. Both ovarian carcinoma antigens expressed epitopes for the anti-human milk fat globule membrane antibodies M18 and 115G2 which were not apparently expressed in the breast carcinoma antigen preparation (Table I). The only other major difference in epitope expression in these three antigen preparations was that the reaction of Ca1 with the ovarian serous cyst adenocarcinoma antigen was much reduced in comparison with its reactivity with the other antigens.

Table II shows the results of further tests examining the reactivity of monoclonal antibodies with the three antigen preparations. No antigen binding was obtained with antibodies reactive against colonic tumours including C154 which binds to a variety of normal and tumour tissues and antibodies C161 and 11.285.14 which react

**Table I** Reactivity of monoclonal antibodies with NCRC-11-defined antigen preparations isolated from ovarian and breast tumours

| | *Mean c.p.m. ± s.d. (– background) bound to NCRC-11 defined antigen isolated from:* | | |
|---|---|---|---|
| *Antibody* | *Ovarian-mucinous adeno-carcinoma* | *Ovarian-serous cyst adeno-carcinoma* | *Breast carcinoma* |
| NCRC-11 | 7,274 ± 116 | 2,920 ± 80 | 7,430 ± 152 |
| HMFG-1 | 9,380 ± 257 | 2,935 ± 215 | 5,333 ± 219 |
| HMFG-2 | 7,485 ± 449 | 3,397 ± 82 | 6,408 ± 151 |
| EMA | 7,786 ± 214 | 3,170 ± 128 | 7,477 ± 383 |
| M8 | 7,176 ± 241 | 3,720 ± 119 | 7,042 ± 180 |
| M18 | 1,980 ± 235 | 655 ± 61 | 18 ± 66 |
| M24 | 117 ± 61 | 100 ± 52 | 154 ± 148 |
| 115D8 | 13,238 ± 305 | 5,659 ± 236 | 13,931 ± 254 |
| 115F5 | 6,343 ± 467 | 3,716 ± 155 | 5,989 ± 158 |
| 115G2 | 2,843 ± 105 | 921 ± 23 | 318 ± 171 |
| Ca1 | 3,867 ± 232 | 317 ± 74 | 4,837 ± 186 |
| Ca2 | 6,954 ± 206 | 2,483 ± 61 | 5,667 ± 139 |
| Ca3 | 5,946 ± 38 | 2,095 ± 116 | 6,743 ± 73 |

**Table II** Reactivity of monoclonal antibodies with NCRC-11-defined antigen preparations isolated from ovarian and breast tumours

| | *Mean c.p.m. ± s.d.(−background) bound to NCRC-11 defined antigen isolated from:* | | |
|---|---|---|---|
| *Antibody* | *Ovarian-mucinous adeno-carcinoma* | *Ovarian-serous cyst adeno-carcinoma* | *Breast carcinoma* |
| NCRC-11 | 6,376±91 | 2,685±51 | 7,343±318 |
| C154 | −7±55 | −130±44 | 61±43 |
| C161 | −15±61 | 22±20 | 39±109 |
| 11.285.14 | 78±36 | −17±84 | −42±86 |
| 791T/36 | −105±17 | −10±43 | 18±30 |
| 791T/48 | 71±23 | 59±15 | −41±98 |
| W6/32 | 391±259 | 173±241 | 140±238 |
| IgM | 39±35 | 58±55 | −53±75 |
| NMS | −104±52 | 162±37 | 148±66 |

with NCA-1 and CEA, respectively. Antibodies against human osteogenic sarcomas (791T/36 and 791T/48) and against HLA-A,B,C (shared determinant) (W6/32), as well as normal mouse IgM and normal mouse serum, failed to bind to any of the antigen preparations.

*Competitive inhibition of $^{125}$I-NCRC-11 antibody binding*

The monoclonal antibodies listed in Table I were assayed for their capacity to inhibit the binding of $^{125}$I-NCRC-11 antibody to the three NCRC-11 antigen preparations adsorbed to the wells of the microtest plates. Both tissue culture supernatants and ascitic fluids were used as the source of antibody. With each individual antibody, and at any antibody dilution tested, its capacity to inhibit the binding of $^{125}$I-NCRC-11 antibody to the 3 antigens was virtually identical (Figures 1 and 2). On the basis of these tests, the antibodies could be divided into several categories with respect to their capacity to inhibit $^{125}$I-NCRC-11 antibody binding to each of the three antigen preparations. Antibodies EMA and M8 were potent inhibitors of $^{125}$I-NCRC-11 antibody binding and they were similar in their inhibitory activity to unlabelled NCRC-11 antibody (Figure 1, panels a and d; Figure 2, panels a and b). Antibodies HMFG-1 and HMFG-2, and to a lesser extent antibodies Ca1, Ca2 and Ca3 partially inhibited $^{125}$I-NCRC-11 antibody binding to the ovarian and breast carcinoma antigens (Figure 1, panels b, c, e, f and g respectively). Essentially, non-inhibitory antibodies include 115D8 and 115F5 (Figure 2, panels e and f) which react strongly with all three NCRC-11 antigen preparations (Table I) as well as antibodies M18, M24 and 115G2 (Figure 2, panel c, d and g respectively) which react weakly or not at all with the ovarian and breast carcinoma antigens (Table I).

*Sandwich immunoassay*

A sandwich immunoassay was employed to confirm the co-expression of monoclonal antibody defined epitopes on the same molecules in each of the three NCRC-11 antigen preparations from ovarian and breast carcinomas. These tests were performed with those antibodies which were strongly reactive with the antigen preparations and which were available as ascitic fluids (using hybridoma supernatants, insufficient antibody is adsorbed to the wells to capture added NCRC-11 antigen detectable with radiolabelled NCRC-11 antibody).

As shown in Table III, $^{125}$I-labelled NCRC-11 antibody bound to wells coated with NCRC-11, 115D8, 115F5 and M8 antibodies and to which the three antigens had been added. No binding was obtained with wells treated with buffer rather than antigen. Thus, in order for $^{125}$I-labelled NCRC-11 antibody to bind to antigen bound to the adsorbed NCRC-11 antibodies (as was the case with each antigen), NCRC-11 epitopes must represent repeated structures on the antigens. Also, for binding of $^{125}$I-labelled NCRC-11 antibody to antigen treated wells which were coated with 115D8, 115F5 or M8 antibodies, then the epitopes

**Table III** Analysis of epitope expression on NCRC-11 defined antigen preparations using a sandwich immunoassay

| | *Binding of $^{125}$I-NCRC-11 antibody to antibody-coated wells treated with NCRC-11 antigen isolated from:* | | |
|---|---|---|---|
| *Wells coated with ascitic fluid (1/500)* | *Ovarian-mucinous adeno-carcinoma* | *Ovarian-serous cyst adeno-carcinoma* | *Breast carcinoma* |
| NCRC-11 | 6,687±94 (−93±43)[a] | 3,362±105 (80±23) | 7,425±464 (76±46) |
| 115D8 | 7,762±179 (−31±8) | 3,155±47 (135±13) | 8,617±489 (163±23) |
| 115F5 | 1,382±271 (−86±35) | 1,065±246 (120±73) | 1,789±128 (147±10) |
| M8 | 1,781±243 (−86±24) | 719±43 (94±13) | 1,859±86 (129±13) |

[a]Figures in parenthesis represent the binding of $^{125}$I-NCRC-11 antibody in the absence of antigen.

**Figure 1** Competitive inhibition of binding of $^{125}$I-NCRC-11 antibody to NCRC-11 defined antigens isolated from ovarian mucinous carcinoma (●——●), ovarian serous cyst carcinoma (■——■), and breast carcinoma (▲——▲). Monoclonal antibodies in hybridoma tissue culture supernatants were examined for their capacity to inhibit $^{125}$I-labelled antibody binding at the dilutions shown.

**Figure 2** Competitive inhibition of binding of $^{125}$I-NCRC-11 antibody to NCRC-11 defined antigens isolated from ovarian mucinous carcinoma (●——●), ovarian serous cyst carcinoma (■——■), and breast carcinoma (▲——▲). Monoclonal antibodies in ascitic fluids (panels a to g) and normal mouse serum (NMS – panel h) were examined for their capacity to inhibit $^{125}$I-NCRC-11 antibody binding at the dilutions shown.

defined by 115D8, 115F5 and M8 antibodies must be co-expressed with NCRC-11 defined epitopes upon at least a proportion of the NCRC-11 antigenic molecules isolated from the three tumour sources.

*Neuraminidase treatment of NCRC-11 antigens*

Desialylation of breast tumour tissue sections with neuraminidase reveals the immunodominant oligosaccharide sequence of the I(Ma) blood group antigen, Gal$\beta$1→4GlcNAc$\beta$1→6-, defined by the monoclonal antibody M18 (Foster & Neville, 1984). The three NCRC-11 defined antigen preparations were treated with neuraminidase and then examined for their capacity to bind the M18 antibody. Untreated and treated antigen preparations were also examined for their capacity to bind the antibodies NCRC-11 and Ca1. Measurement of the effect of neuraminidase on Ca1 binding was included as a positive control for the enzyme digestion, since the epitope for Ca1 involves sialic acid (Bramwell *et al.*, 1985). As shown in Figure 3, neuraminidase treatment of NCRC-11 antigens from ovarian and breast carcinomas did not modify their capacity to bind NCRC-11 antibody, whereas the binding of Ca1 to the treated antigens was virtually abolished. Conversely, the binding of the M18 antibody was considerably increased with the neuraminidase treated antigens. This is most clearly shown with NCRC-11 antigen from breast carcinoma – the M18 antibody failed to bind to the untreated antigen (Table I and Figure 3c) whereas its binding to neuraminidase treated antigen was 64% of the level of NCRC-11 antibody binding (Figure 3c).

**Figure 3** NCRC-11, Ca1 and M18 binding to neuraminidase treated NCRC-11 antigen preparations isolated from (a) ovarian mucinous carcinoma, (b) ovarian serous cyst carcinoma, and (c) breast carcinoma. Antigen preparations were adsorbed to the wells of microtest plates and treated for 60 min at 37°C with control buffer solutions or neuraminidase (*Clostridium perfringens* Type V neuraminidase at 100 mU ml$^{-1}$ in 200 mM sodium acetate buffer, pH 5.5 containing 1 mM phenyl methyl sulphonyl fluoride, PMSF). Open bars represent antibody binding after treatment with washing buffer, hatched bars represent antibody binding after treatment with sodium acetate/PMSF buffer and solid bars show antibody binding after neuraminidase treatment. After buffer or enzyme treatment, the plates were washed 4 times and blocked with washing buffer for 1 h before the addition of antibodies. Error bars represent s.d.

### Discussion

The three antigen preparations described in this report were isolated from the two major types of malignant ovarian carcinoma (mucinous and serous) and from breast carcinoma. The antigen preparations were purified by their binding to, and elution from, Sepharose-linked NCRC-11 antibodies, so that all molecules in each antigen preparation should express the NCRC-11 epitopes. By normalizing the data in Table I, so that the reactivity of NCRC-11 antibody with each antigen is equalized, a profile of antibody reactivity with the three antigens may be constructed. As shown in Figure 4 this type of epitope profile illustrates the remarkable similarity in the three antigen preparations with respect to their reactivity with the antibodies tested. Of note, the antibody Ca1 displayed the most variability in expression between the three antigen preparations. This may be due to variability in the degree of sialylation of the three antigens since the Ca1 epitope involves sialic acid (Bramwell *et al.*, 1985). Neuraminidase treatment of antigens abolished Ca1 binding activity but clearly resulted in an increase in the binding of the M18 antibody (Figure 3). In this case, the excess sialylation of the I(Ma) blood group antigen, defined by the M18 antibody, leaves this determinant in a cryptic or masked form.

**Figure 4** Profile of monoclonal antibody binding to NCRC-11-defined antigens from ovarian mucinous carcinoma, ovarian serous cyst carcinoma and breast carcinoma.

Tests examining the capacity of various antibodies to inhibit the binding of $^{125}$I-NCRC-11 antibody to the three antigen preparations were performed. With any one of the antibodies tested the inhibition curves with the three antigens were co-incidental (Figures 1 and 2). This infers that the topographical disposition of epitopes defined by any one of the antibodies tested, in relation to epitopes defined by the NCRC-11 antibody, is the same or remarkably similar in each of the three antigen preparations. The degree of inhibition of binding of $^{125}$I-NCRC-11 antibody to antigen (Figures 1 and 2) was not related to the magnitude of the reaction of that antibody with the antigen. Thus, the 115D8 antibody, which reacts strongly with each antigen (Table I; Figure 4) failed to inhibit or modify $^{125}$I-NCRC-11 antibody binding (Figure 2). Sandwich immunoassays confirmed that NCRC-11 and 115D8 epitopes are indeed co-expressed on the same molecules (Table III).

The NCRC-11 defined antigens as isolated from ovarian and breast tumours, clearly express a number of important epitopes defined by several well characterized monoclonal antibodies. It is of interest that antibody binding reactions to these various epitopes have been exploited for diverse purposes in relation to breast cancer and malignant ovarian tumours. Taking a number of examples, NCRC-11 antibody binding to tissue sections is of prognostic significance in breast cancer (Ellis *et al.*, 1985) although HMFG-1 and HMFG-2 were of less value in similar studies (Wilkinson *et al.*, 1984; Berry *et al.*, 1985). Antibodies like those of the Ca series (Bramwell *et al.*, 1985) have found application as markers of malignant cells in diagnostic cytological tests. On the other hand, the 115D8 antibody has been utilized for the development of a sandwich immunoassay for the determination of its target antigen (the so-called MAM-6 antigen) in the circulation of breast cancer patients, and MAM-6 antigen levels correlated with tumour load (Hilkens *et al.*, 1985). Thus, this particular antibody has important diagnostic applications.

The value of these antibodies reactive with NCRC-11 antigen and related high molecular weight mucins, for targeting agents to tumours would be expected to be of limited value because of the wide distribution of the relevant epitopes on normal tissues. However, HMFG-1 and HMFG-2 antibodies have been employed for the radio-diagnostic imaging of ovarian, breast and gastrointestinal tumours (Epenetos *et al.*, 1982) and for the staging of cervical cancer by antibody (HMFG-2) guided lymphangiography (Epenetos, 1985). In addition, therapeutic doses of $^{131}$I linked to HMFG-2 antibodies have been administered to patients with malignant effusions with encouraging results (Epenetos, 1984).

These studies were supported by the Cancer Research Campaign. Sincere thanks are expressed to the following for providing samples of their antibodies: to Drs. J. Taylor-Papadimitriou and J. Burchell for HMFG-1 and HMFG-2; to Drs. J. Hilgers and J. Hilkens for 115D8, 115F5 and 115G2; to Dr. P.A.W. Edwards for M8, M18 and M24; to Drs. M.E. Bramwell and W.D. Smith and Professor H. Harris for Ca1, Ca2 and Ca3 and to Dr. G.F. Rowland for 11.285.14. Mrs. H. Beverley-Clarke is thanked for providing purified preparations of the NCRC-11 antibody.

The assistance of Mrs. M. Trevers in the preparation of this manuscript is gratefully acknowledged.

### References

ASHALL, F., BRAMWELL, M.E. & HARRIS, H. (1982). A new marker for human cancer cells. 1. The Ca antigen and the Ca1 antibody. *Lancet*, **ii**, 1.

BERRY, N., JONES, D.B., SMALLWOOD, J., TAYLOR, I., KIRKHAM, N. & TAYLOR-PAPADIMITRIOU, J. (1985). The prognostic value of the monoclonal antibodies HMFG-1 and HMFG-2 in breast cancer. *Br. J. Cancer*, **51**, 179.

BLASZCZYK, M., PAK, K.Y., HERLYN, M. & 4 others (1984). Characterization of gastrointestinal tumor-associated carcinoembryonic antigen-related antigens defined by monoclonal antibodies. *Cancer Res.*, **44**, 245.

BRAMWELL, M.E., BHAVANANDAN, V.P., WISEMAN, G. & HARRIS, H. (1983). Structure and function of the Ca antigen. *Br. J. Cancer*, **48**, 177.

BRAMWELL, M.E., GHOSH, A.K., SMITH, W.D., WISEMAN, S., SPRIGGS, A. & HARRIS, H. (1985). Ca2 and Ca3. New monoclonal antibodies as tumor markers in serous effusions. *Cancer*, **56**, 105.

CORVALAN, J.R.F., AXTON, C.A., BRANDON, D.R., SMITH, W. & WOODHOUSE, C. (1984). Classification of anti-CEA monoclonal antibodies. In *Protides of the Biological Fluids*, Peeters, H. (ed) **31**, p. 921, Pergamon Press, Oxford.

EDWARDS, P.A.W. & BROOKS, I.M. (1984). Antigenic subsets of human breast epithelial cells distinguished by monoclonal antibodies. *J. Histochem. Cytochem.*, **32**, 531.

ELLIS, I.O., ROBINS, R.A., ELSTON, C.W., BLAMEY, R.W., FERRY, B. & BALDWIN, R.W. (1984). A monoclonal antibody, NCRC-11, raised to human breast carcinoma. I. Production and immunohistological characterization. *Histopathology*, **8**, 501.

ELLIS, I.O., HINTON, C.P., MACNAY, J. & 6 others (1985). Immunocytochemical staining of breast carcinoma with the monoclonal antibody NCRC-11 – A new prognostic indicator. *Br. Med. J.*, **290**, 881.

EMBLETON, M.J., GUNN, B., BYERS, V.S. & BALDWIN, R.W. (1981). Antitumour reactions of monoclonal antibody against a human oesteogenic sarcoma cell line. *Br. J. Cancer*, **43**, 582.

EPENETOS, A.A. (1984). Antibody-guided irradiation of malignant lesions: Three cases illustrating a new method of treatment – A report from the Hammersmith Oncology Group and the Imperial Cancer Research Fund. *Lancet*, **i**, 1441.

EPENETOS, A.A. (1985). Antibody guided lymphangiography in the staging of cervical cancer. *Br. J. Cancer*, **51**, 805.

EPENETOS, A.A., BRITTON, K.E., MATHER, S. & 8 others (1982). Targeting of iodine-123-labelled tumour-associated monoclonal antibodies to ovarian, breast and gastrointestinal tumours. *Lancet*, **ii**, 999.

FOSTER, C.S. & NEVILLE, A.M. (1984). Monoclonal antibodies to the human mammary gland. III. Monoclonal antibody LICR-LON-M18 identifies impaired expression and excess sialylation of the I(Ma) cell surface antigen by primary breast carcinoma cells. *Human Pathol.*, **15**, 502.

FRAKER, P.J. & SPECK, J.C. (1978). Protein and cell membrane iodinations with a sparingly soluble chloramide, 1,3,4,6-tetrachloro-3α, 6α-diphenylglycouril. *Biochem. Biophys. Res. Comm.*, **80**, 849.

HEYDERMAN, E., STRUDLEY, I., POWELL, G., RICHARDSON, T.C., CORDELL, J.L. & MASON, D.Y. (1985). A new monoclonal antibody to epithelial membrane antigen (EMA)-E29. A comparison of its immunocytochemical reactivity with polyclonal anti-EMA antibodies and with another monoclonal antibody, HMFG-2. *Br. J. Cancer*, **52**, 355.

HILKENS, J., BUIJS, F., HILGERS, J., HAGEMAN, Ph., CALAFAT, J., SONNENBERG, A. & VAN DER VALK, M. (1984). Monoclonal antibodies against human milk fat globule membranes detecting differentiation antigens of the mammary gland and its tumours. *Int. J. Cancer*, **34**, 197.

HILKENS, J., KROEZEN, V., BUIJS, F. & 5 others (1985). MAM-6, A carcinoma associated marker: preliminary characterization and detection in sera of breast cancer patients. In: *Proceedings of the International Workshop on Monoclonal Antibodies and Breast Cancer*, Ceriani, R.L. (ed) p. 28. Martinus Nijhoff.

JENSENIUS, J.C. & WILLIAMS, A.F. (1974). The binding of anti-immunoglobulin antibodies to rat thymocytes and thoracic duct lymphocytes. *Eur. J. Immunol.*, **4**, 91.

LOWRY, O.H., ROSEBROUGH, N.J., FARR, A.L. & RANDALL, R.J. (1951). Protein measurement with the Folin phenol reagent. *J. Biol. Chem.*, **193**, 265.

ORMEROD, M.G., McILHINNEY, J., STEELE, K. & SHIMIZU, M. (1985). Glycoprotein PAS-O from the human milk fat globule membrane carries antigenic determinants for epithelial membrane antigen. *Molec. Immunol.*, **22**, 265.

PRICE, M.R. & BALDWIN, R.W. (1974). Preparation of aminoazo dye induced rat heptatoma membrane fractions retaining tumour specific antigen. *Br. J. Cancer*, **30**, 382.

PRICE, M.R., EDWARDS, S., OWAINATI, A. & 4 others (1985). Multiple epitopes on a human breast carcinoma associated antigen. *Int. J. Cancer.*, **36**, 567.

PRICE, M.R., EDWARDS, S. & BALDWIN, R.W. (1986). Application of a subcellular membrane-antibody binding assay for the analysis of antigen expression in human tumours. *J. Cancer Res. Clin. Oncol.*, **111**, 169.

SHIMIZU, M. & YAMAUCHI, K. (1982). Isolation and characterization of mucin-like glycoprotein in human milk fat globule membrane. *J. Biochem.*, **91**, 515.

TAYLOR-PAPADIMITRIOU, J., PETERSON, J., ARKLIE, J., BURCHELL, J., CERIANI, R.L. & BODMER, W.F. (1981). Monoclonal antibodies to epithelium-specific components of the human milk fat globule membrane: production and reaction with cells in culture. *Int. J. Cancer*, **28**, 17.

TOWBIN, H., STAEHELIN, T. & GORDON, J. (1979). Electrophoretic transfer of proteins from polyacrylamide gels to nitrocellulose sheets: procedure and some applications. *Proc. Nat. Acad. Sci., USA.*, **76**, 4350.

WILKINSON, M.J.S., HOWELL, A., HARRIS, M., TAYLOR-PAPADIMITRIOU, J., SWINDELL, R. & SELLWOOD, R.A. (1984). The prognostic significance of two epithelial membrane antigens expressed by human mammary carcinomas. *Int. J. Cancer*, **33**, 299.